# Proteomic landscape of SARS-CoV-2– and MERS-CoV–infected primary human renal epithelial cells

**DOI:** 10.26508/lsa.202201371

**Published:** 2022-02-02

**Authors:** Aneesha Kohli, Lucie Sauerhering, Sarah K Fehling, Kevin Klann, Helmut Geiger, Stephan Becker, Benjamin Koch, Patrick C Baer, Thomas Strecker, Christian Münch

**Affiliations:** 1 Institute of Biochemistry II, Faculty of Medicine, Goethe University, Frankfurt am Main, Germany; 2 Institute of Virology, Philipps University Marburg, Marburg, Germany; 3 German Center for Infection Research (DZIF), Partner Sites Gieβen-Marburg-Langen, Marburg, Germany; 4 Division of Nephrology, Department of Internal Medicine III, University Hospital, Goethe-University, Frankfurt am Main, Germany; 5 Frankfurt Cancer Institute, Frankfurt am Main, Germany; 6 Cardio-Pulmonary Institute, Frankfurt am Main, Germany

## Abstract

Acute kidney injury is associated with mortality in COVID-19 patients. However, host cell changes underlying infection of renal cells with SARS-CoV-2 remain unknown and prevent understanding of the molecular mechanisms that may contribute to renal pathology. Here, we carried out quantitative translatome and whole-cell proteomics analyses of primary renal proximal and distal tubular epithelial cells derived from human donors infected with SARS-CoV-2 or MERS-CoV to disseminate virus and cell type–specific changes over time. Our findings revealed shared pathways modified upon infection with both viruses, as well as SARS-CoV-2-specific host cell modulation driving key changes in innate immune activation and cellular protein quality control. Notably, MERS-CoV infection–induced specific changes in mitochondrial biology that were not observed in response to SARS-CoV-2 infection. Furthermore, we identified extensive modulation in pathways associated with kidney failure that changed in a virus- and cell type–specific manner. In summary, we provide an overview of the effects of SARS-CoV-2 or MERS-CoV infection on primary renal epithelial cells revealing key pathways that may be essential for viral replication.

## Abstract (precis)

Translatome and proteome analyses of human proximal and distal tubular cells during coronavirus infection reveal distinctive host cell response patterns important for viral replication and renal pathology.

## Introduction

Since the dawn of the 21^st^ century, the pathogenicity of coronaviruses has been affecting the world every few years through highly human-to-human transmissible viruses such as severe acute respiratory syndrome coronavirus (SARS-CoV) and Middle East respiratory syndrome coronavirus (MERS-CoV) (1). In 2019, the novel coronavirus SARS-CoV-2 emerged, resulting in the COVID-19 pandemic that continues to affect millions across the globe, with multi-organ failure being the most frequent cause of severe illness and death (2, 3). Besides lung and heart failure, kidney injury is among the predominant terminal organ failures in intensive care patients (2, 4). Importantly, acute kidney injury (AKI) is reported in up to 78% of critically ill COVID-19 patients (5) and up to 90% in those who require mechanical ventilation (6). AKI in SARS-CoV-2–infected patients is associated with increased morbidity and mortality, necessitating further research to understand the link between AKI and COVID-19 and the underlying mechanisms (5, 6, 7, 8). Originally thought to be a primarily pulmonary disease, in the recent months, extrapulmonary tissues have been shown to be targeted by SARS-CoV-2 during the systemic phase of COVID-19 (9).

Infection takes place by SARS-CoV-2’s established tropism for angiotensin-converting enzyme 2 (ACE-2) (10, 11), which shows high expression in human renal tissue (12). In addition, SARS-CoV-2 likely uses additional cell surface molecules for virus entry into human cells including kidney-specific host factors (13, 14, 15). Essentially, SARS-CoV-2 has been detected in renal tubular epithelial cells, showing a strong staining for virus antigen and/or viral particles by electron microscopy (16, 17). The poor prognosis associated with renal pathology has also previously been observed in patients infected with SARS-CoV and MERS-CoV (1, 18). Acute respiratory distress syndrome (ARDS) is one of the foremost clinical manifestations in coronavirus infections. However, the impact on kidneys is also dominant and was recently explained by the lung–kidney axis (19).

MERS-CoV is known to replicate 1,000-fold more effectively in renal epithelial cells in comparison to bronchial epithelial cells (20). Human autopsy data showed the presence of MERS-CoV in renal epithelial cells (21) and renal failure as a severe medical condition has been reported in up to 75% of patients infected with MERS-CoV, showing the need for renal replacement therapy in critically ill patients (20, 22). Furthermore, viral RNA of both SARS-CoV-2 and MERS-CoV have been recovered from patient urine; and viable virus isolation has also been reported for SARS-CoV-2 suggesting renal viral replication in vivo (23, 24, 25). Whereas MERS-CoV presents a uniquely high incidence of renal failure, this was less frequently observed in SARS-CoV patients; though renal impairment was associated with higher mortality (18, 26).

Despite the substantial co-occurrence of AKI in critical MERS-CoV and COVID-19 patients, the understanding of coronavirus-associated renal pathology remains limited, especially in light of the novel SARS-CoV-2. Moreover, there is also a lack of comprehensive proteomic studies in primary cells to study the similarities and differences between coronaviruses and their impact on renal pathology. To fill this gap in our knowledge, we performed infectivity analysis of SARS-CoV-2 and MERS-CoV in both proximal and distal tubular epithelial cells that are known to respond differently to infection (17) and further compared the infection-induced translatome and proteome changes across viruses and cell types. We used primary human renal proximal and distal tubular cells (PTC and DTC, respectively) that highly resemble cell type–specific expression patterns observed in vivo (27, 28, 29). We monitored different time points post-infection and identified key determinants of the host cell response to coronavirus infection. Our findings show common and virus-specific pathways with relevance for immune activation differences at the level of both temporal and intensity scales, changes in the mitochondrial proteome profile and identify significant alterations in both mitochondrial and nuclear pore factors as potential contributors of renal pathology. Better knowledge of these pathways may offer new opportunities for the design of novel treatment options against highly pathogenic coronaviruses.

## Results

### Human proximal and distal tubular epithelial cells are susceptible to infection by SARS-CoV-2 or MERS-CoV

**Figure 1.**
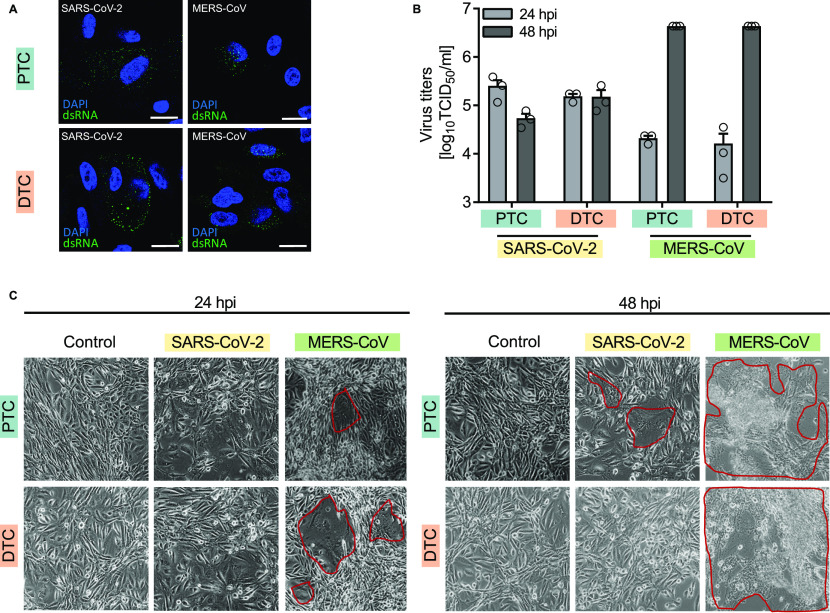
Infection of primary human renal cells with SARS-CoV-2 and MERS-CoV. **(A)** SARS-CoV-2 or MERS-CoV infection of proximal and distal renal tubular epithelial cells (PTC and DTC, respectively) grown in chamber slides. For immunofluorescence analysis, cells were stained with a monoclonal anti-dsRNA antibody at 24 hpi. Nuclei were visualized by DAPI staining. Scale bars, 20 μm. **(B)** Growth kinetics of SARS-CoV-2 or MERS-CoV in PTC or DTC. Viral titers were measured by TCID_50_ analysis at indicated time points. **(C)** Cytopathic effects and syncytia formation in PTC and DTC infected with SARS-CoV-2 or MERS-CoV was documented in live cells by phase contrast microscopy at a magnification of ×100 at 24 and 48 hpi. Red lines indicate virus-induced foci formation. Representative images of three independent biological replicates are shown.

**Figure S1.**
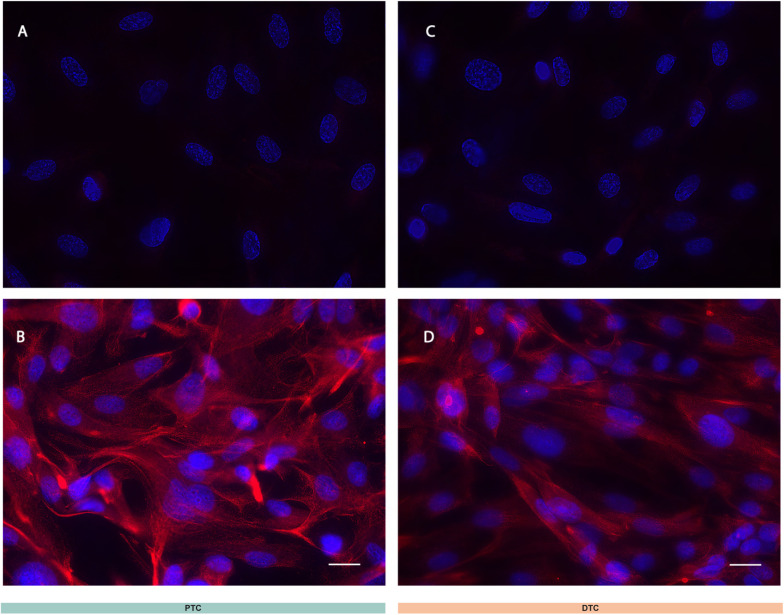
ACE-2 protein expression in renal tubular epithelial cells. **(A, B, C, D)** ACE-2 immunofluorescence staining in cultured PTC (A, B) and DTC (C, D). Representative image (from two biological replicates). **(B, D)** ACE-2 staining (red) was performed using primary antibody anti-ACE-2 and a Cy3-conjugated secondary antibody. **(A, C)** Controls of nonspecific fluorescence were performed on cells processed without the primary antibody. Images were taken with a Keyence BZ-X800 microscope. Nuclei were counterstained with DAPI (blue). Scale bars, 20 μm.

**Figure S2.**
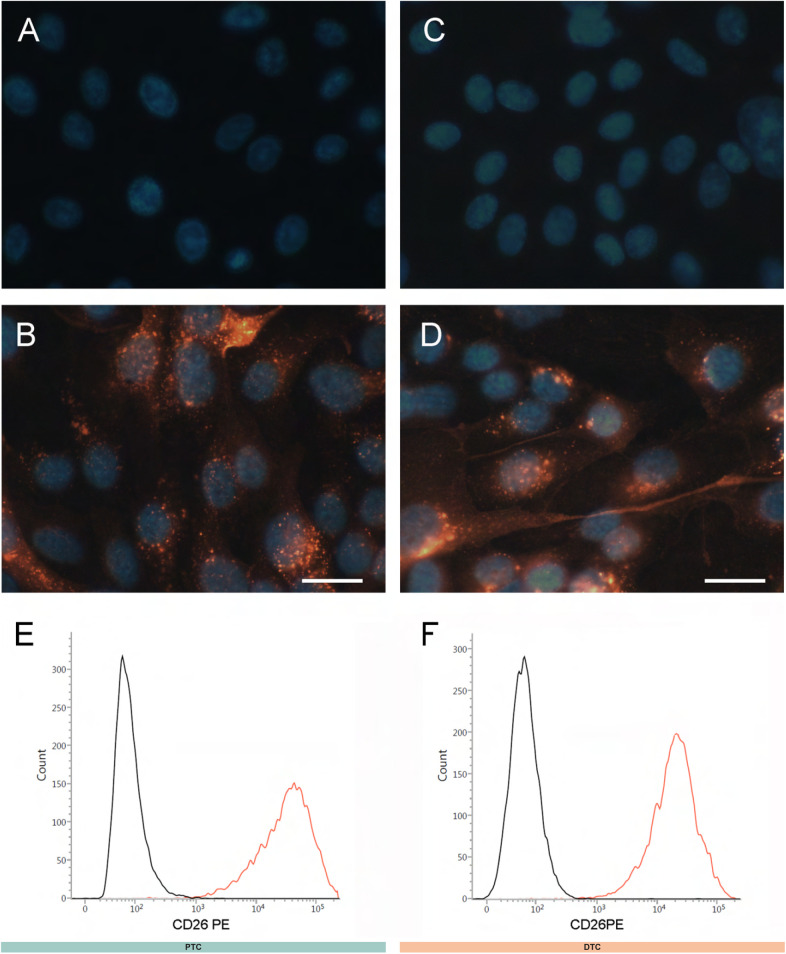
DPP4 protein expression in renal tubular epithelial cells. **(A, B, C, D)** DPP4 (CD26) immunofluorescence staining in cultured PTC (A, B), and DTC (C, D). Representative image (from two biological replicates). **(B, D)** DPP4 staining (red) was performed using a PE-labeled antibody anti-CD26. **(A, C)** Controls of nonspecific fluorescence were performed on cells processed without the antibody. Nuclei were counterstained with DAPI (blue). Images were taken with a Zeiss Axiolab microscope. Scale bars, 20 μm. **(E, F)** Representative flow cytometric overlay histograms of DPP4 expression (red) in PTC (E) and DTC (F) compared with isotype-matched controls (black).

To demonstrate susceptibility of primary human renal proximal (PTC) and distal (DTC) tubular epithelial cells for SARS-CoV-2 or MERS-CoV infection, cells were grown in chamber slides and infected at an MOI of 0.01 to limit early onset of cytopathic effects and apoptosis (30). At 24 h post-infection (hpi), virus-infected cells were visualized by staining of dsRNA, an intermediate structure during genome replication (Fig 1A). Immunofluorescence staining confirmed the expression of the SARS-CoV-2 and MERS-CoV entry receptors ACE-2 and dipeptidyl-peptidase 4 (DPP4) (31), respectively, in PTC and DTC (Figs S1A–D and S2A–F).

Next, PTC and DTC were grown in 12-well plates and infected with SARS-CoV-2 or MERS-CoV to establish conditions for proteomics analyses. Growth kinetics as well as monitoring of cytopathic effects (CPE) were performed in parallel and showed productive viral infection in both cell types (Fig 1B and C). However, at 48 hpi, viral titers in the culture supernatants of SARS-CoV-2–infected cells were lower in comparison with the viral titers measured for MERS-CoV (Fig 1B). In addition, CPE were also comparatively less pronounced in cells infected with SARS-CoV-2 (Fig 1C).

### Global landscape of translatome and proteome changes upon infection of PTC and DTC with SARS-CoV-2 or MERS-CoV

**Figure 2.**
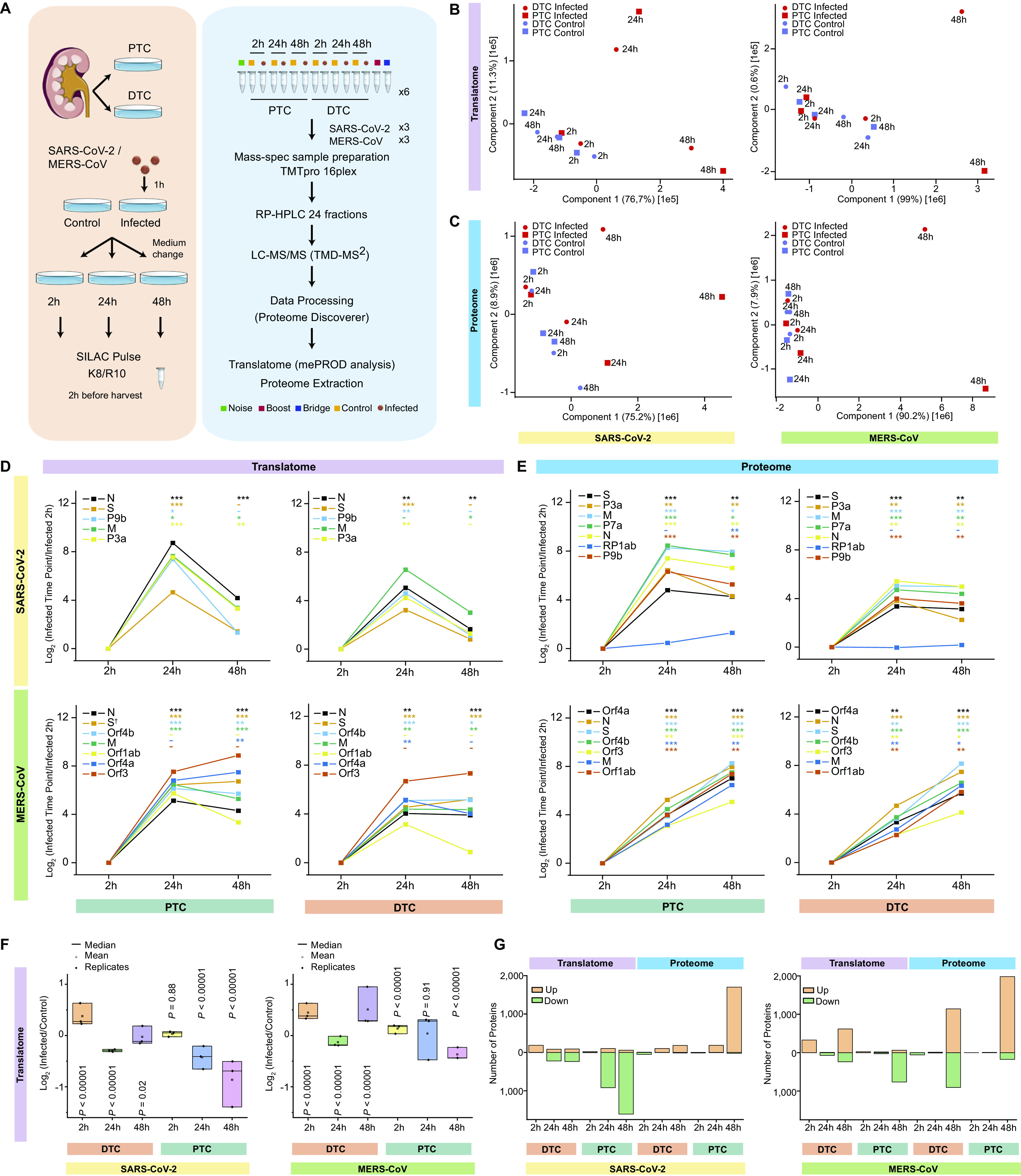
Global proteome and translatome landscapes of SARS-CoV-2 or MERS-CoV–infected primary human renal epithelial cells. **(A)** Experimental design for global translatome and proteome quantification upon viral infection at 2, 24, and 48 hpi. Renal tubular epithelial cells—proximal (PTC) and distal (DTC)—were infected with SARS-CoV-2 or MERS-CoV at an MOI of 0.01 for 1 h, medium changed, and cells switched to heavy stable isotope labeling by amino acids in cell culture (SILAC) medium for 2 h before harvest along with corresponding controls. Samples were labeled with 16plex tandem mass tag (TMT) and spread across six plexes for liquid chromatography-tandem mass spectrometry measured with targeted mass difference (TMD) method. Proteome Discoverer 2.4 and in-house python scripts were used for data analysis. K8: ^13^C_6_,^15^N_2_ L-lysine; R10: ^13^C_6_,^15^N_4_ L-arginine. **(B, C)** Principal component analysis of translatome (B) and proteome (C) data (n = 3 independent biological replicates). **(D, E)** For all detected viral proteins, translation rates (D) and proteome (E) levels over time are depicted as the mean log_2_ fold-changes with respect to their corresponding 2 hpi group (n = 3 independent biological replicates). *: The 2 hpi value could not be quantified and the 2 hpi control was used instead. *t* test significance is indicated as ***P-value < 0.001; **P-value < 0.01; *P-value < 0.05; -: not significant. **(F)** Comparison of global translation distribution across time points. Data represented as the mean log_2_ fold-change across all quantified proteins per replicate. Circles represent means across all proteins of one replicate, squares indicate mean values across replicates and lines indicate median values across replicates (n = 3 independent biological replicates). Significance testing was done using one-sample *t* test assuming normal distribution of data with null hypothesis of mean = 0. Small *P*-values (*P*-values < 1.00 × 10^−80^) were rounded up to *P* < 0.00001. **(G)** Number of significantly changed proteins (−0.5 ≥ log_2_ fold-change ≥ 0.5 and *P*-value ≤ 0.05) in the translatome and proteome across time points, cell types, and viruses.

**Figure S3.**
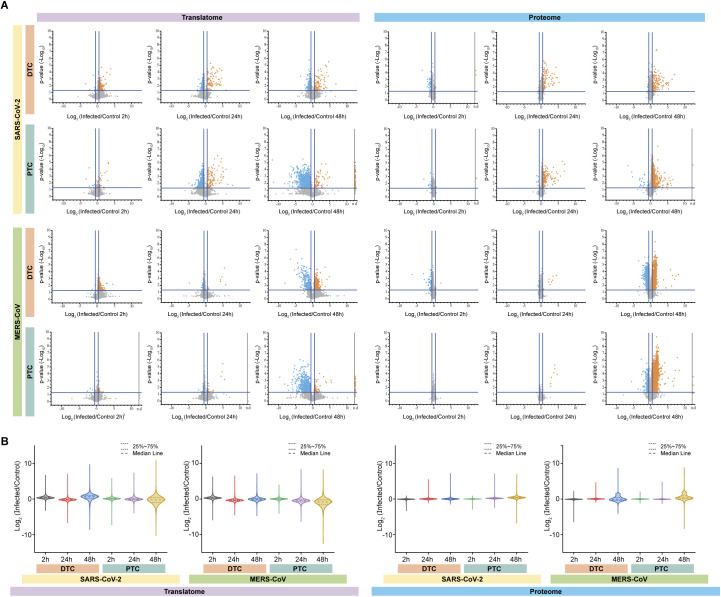
Global changes in proteome and translatome upon SARS-CoV-2- or MERS-CoV infection of primary human renal epithelial cells. **(A)** Volcano plots showing changes in protein distributions across time points, cell types and viruses. Data are represented as log_2_ fold-changes for mean protein abundance upon infection compared with controls (n = 3 independent biological replicates). *P*-values were calculated using two-sided unpaired *t* test with equal variance assumed. Proteins significantly increased or decreased in abundance upon infection are highlighted in orange and blue, respectively. n.d, not defined. **(B)** Global translatome and proteome distribution for all detected proteins across time points, cell type and viruses are represented with kernel smoothing and Silverman bandwidth.

To obtain an unbiased profile of the cellular response to SARS-CoV-2 and MERS-CoV infection in human primary renal tubular epithelial cells, we set up quantitative translatome and proteome proteomics of in vitro infected cells over time (Fig 2A). Cells were infected at an MOI of 0.01 to optimise the temporal study of early and late host cell responses across three different time points – 2, 24 and 48 hpi. We then pulsed-SILAC labeled cells for 2 h using the previously described mePROD method (32, 33) to allow translatome and proteome measurements across control and infected cells at different times post-infection (all in triplicate, total of 72 samples). Samples were multiplexed using tandem mass tags (TMT) and pooled into six multiplexes that were each fractionated into 24 fractions for analysis by LC-MS/MS using targeted mass difference (34). We quantified 5,321 and 5,080 newly synthesized proteins (translatome) upon SARS-CoV-2 and MERS-CoV infection, respectively, and 6,134 and 5,612 proteins at the proteome level, respectively, across all 72 samples (Table S1). Principal component analysis (PCA) of the translatome revealed that SARS-CoV-2–infected PTC and DTC were distinct from non-infected control cells at 24 hpi, with more pronounced effects being observed after 48 hpi (Figs 2B and S3A). This finding was different from MERS-CoV–infected cells that showed first translatomic differences at 48 hpi in comparison to uninfected control cells. These differences were also reflected in our proteome analyses. However, at 24 hpi, only SARS-CoV-2–infected PTC exhibited changes, whereas both PTC and DTC infected with either virus were changed at 48 hpi, with slightly more pronounced effects in PTC than DTC (Figs 2C and S3A).

Table S1 Data for proteome and translatome quantifications for SARS-CoV-2 and MERS-CoV–infected PTC and DTC with their respective controls.

To evaluate viral protein levels over time and to gain insight into the mechanisms potentially explaining the observed differences between the different renal tubular epithelial cell types and viruses, we next examined the temporal profiles of all quantified viral proteins (Fig 2D and E). Our translatome analyses consistently revealed increased translation of viral proteins in PTC when compared with DTC (Fig 2D), which is in agreement with our observation of similarly elevated viral protein levels in PTC (Fig 2E), potentially explaining the observed differences and earlier changes seen in the host cell driven PCA analyses (Fig 2B and C). At 24 hpi, average viral translation rates showed no alteration across viruses (Fig 2D and E). Hence, the differences in the host cell response did not correlate with a delayed infection or synthesis of viral proteins (Fig 2B and C). Notably, we observed a substantial decrease in translation rates for SARS-CoV-2 when comparing infected cells at 24 and 48 hpi, respectively (Fig 2D), resulting in a plateau of SARS-CoV-2 protein levels (Fig 2E). This was in contrast to MERS-CoV protein levels that increased over time, consistent with maintained translation rates (Fig 2E). Overall, we observed comparable viral protein expression levels across different cells and viruses.

We next examined the effects of viral infection on the different host cells. Globally, host cell translation decreased 65% or 39% in PTC and 12% or 8% in DTC after infection with SARS-CoV-2 or MERS-CoV, respectively (Figs 2F and S3B). Thus, observed effects were more pronounced in SARS-CoV-2– versus MERS-CoV–infected cells. The renal tubular epithelial cell type mainly defined translatome differences with PTC showing an approximately fivefold stronger reduction in translation for both SARS-CoV-2 and MERS-CoV infection when compared with DTC. This observation was also reflected by individual host protein levels with an extensive number of proteins exhibiting significantly (−0.5 ≥ log_2_ fold-change ≥ 0.5 and *P*-value ≤ 0.05) reduced translation rates, particularly in PTC (Fig 2G). At the proteome level, we observed a larger number of host proteins that were significantly increased, particularly in PTC (Fig 2G). These data suggest host cell or virus-specific differential alterations of the global cellular responses, resulting in distinctive changes that may be associated with protein accumulation and translation, respectively.

Notably, DTC infected with MERS-CoV consistently responded in a different manner at 48 hpi when compared with other conditions, with global translation rates increased and the proteome showing an extensive number of individual proteins with reduced protein levels (Fig 2F and G). These results suggested that PTC and DTC exhibit distinct signatures and virus-specific host cell responses upon SARS-CoV-2 or MERS-CoV infection, respectively.

Overall, our analyses revealed that PTC were more severely affected by viral infection than DTC and that SARS-CoV-2 infection resulted in an accelerated host cell remodeling when compared with MERS-CoV.

### SARS-CoV-2 and MERS-CoV infections elicit both unique and shared global pathway enrichments across different primary renal tubular epithelial cells

**Figure 3.**
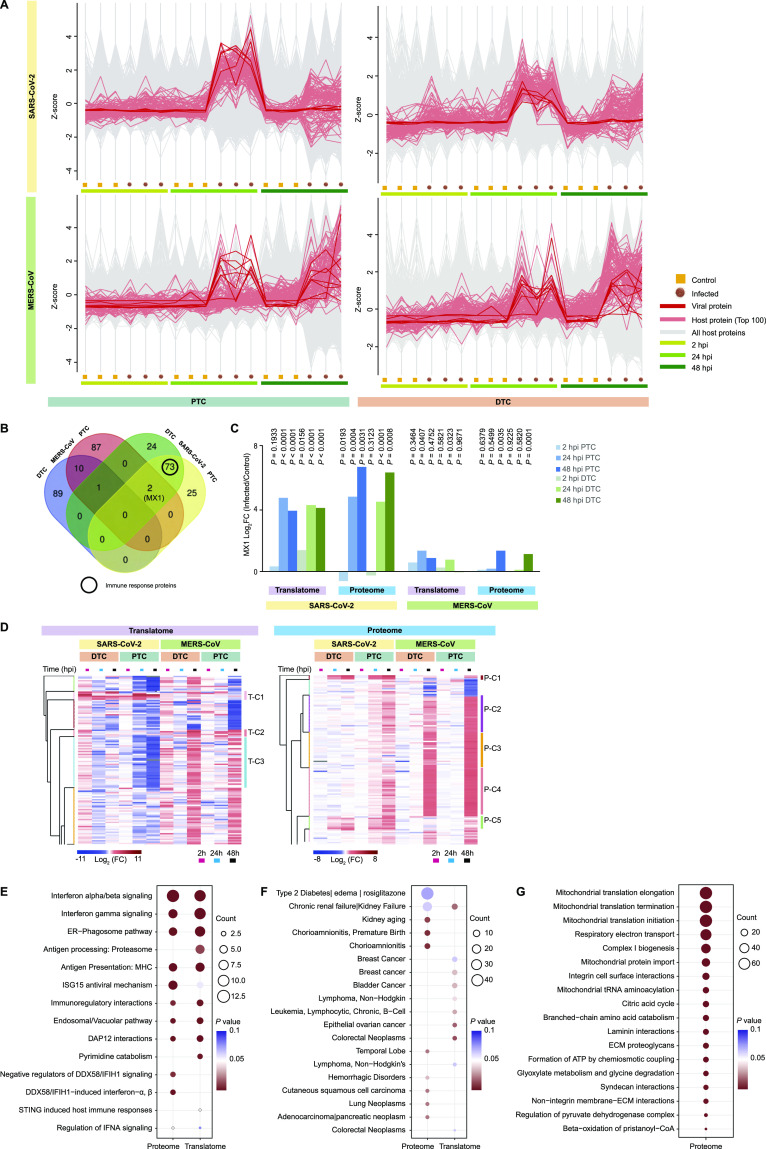
Host responses to SARS-CoV-2 or MERS-CoV infection. **(A)** Top 100 host proteins following average viral translation and proteome level profiles over time. Average profiles were calculated using the Z-scored mean abundance of all detected viral proteins. Individual host protein profiles were compared with the average viral profile and scored using Pearson correlation. **(B)** Venn diagram showing the number of overlapping proteins from the top 100 proteins list for each cell type and viral infection. SARS-CoV-2–infected PTC and DTC show 75 shared proteins predominantly associated with immune response. **(C)** MX1 protein profile across different time points post-infection of PTC and DTC infected either with SARS-CoV-2 or MERS-CoV. Represented are log_2_ fold-changes and corresponding *P*-values (n = 3 independent biological replicates). Small *P*-values were rounded up to *P* < 0.0001. Exact *P*-values are shown in Table S1. **(D)** Row-wise hierarchical clustering analysis for host proteins detected in both viral infections for translatome (left) and proteome (right). Selected clusters are indicated (for complete clustering refer to Fig S4). Each row represents the mean log_2_ fold-change (FC) of one protein upon infection compared with control. **(E, F, G)** Pathway enrichment analyses using DAVID web-tool (see Table S3). **(E)** Reactome pathways analysis for cluster 1 (T-C1 and P-C1) proteins in translatome and proteome. Proteins in this cluster are primarily increased upon SARS-CoV-2 infection. Pathways with *P*-value ≤ 0.05 in either subset are shown. **(F)** Disease pathway analysis for cluster 2 (T-C2 and P-C2), defined by increased translation or protein abundance upon MERS-CoV infection. **(G)** Reactome pathway analysis for proteins belonging to proteome clusters P-C2, P-C3 and P-C4. This subset of proteins was distinctively increased in MERS-CoV–infected PTC and DTC as well as SARS-CoV-2–infected PTC. Pathways with false discovery rate <0.05 are shown.

The detection of viral proteins can result in the immediate activation of various host cell responses, including antiviral signaling pathways. To study potential cell type–specific differences in the host cell response to SARS-CoV-2 or MERS-CoV infection, we determined the top 100 host cell proteins that follow the viral protein translation profile using the average profile of Z-scores from all identified virus-encoded proteins (Fig 3A and Table S2). Interestingly, host cell proteins significantly correlated with the viral protein profile for SARS-CoV-2 but not MERS-CoV–infected cells. When we assessed the determined host cell proteins, we observed a large degree of variation between PTC and DTC in MERS-CoV–infected cells (Fig 3B). In contrast, SARS-CoV-2–infected PTC and DTC revealed an extensive overlap of responding host proteins (75 of 100 proteins of which 41 were significantly correlated with a false discovery rate < 0.05) that were predominantly part of the immune response (Fig 3B). Notably, we found proteins PARP9 and MX1 to be shared across SARS-CoV-2–infected PTC and DTC as well as MERS-CoV–infected PTC. Particularly, the IFN-induced GTP-binding antiviral protein MX1 was significantly elevated at both translation and proteome levels for all conditions for at least one time point post-infection (Fig. 3C). Interestingly, elevated *MX1* levels were reported in COVID-19 patients (35).

**Figure 4.**
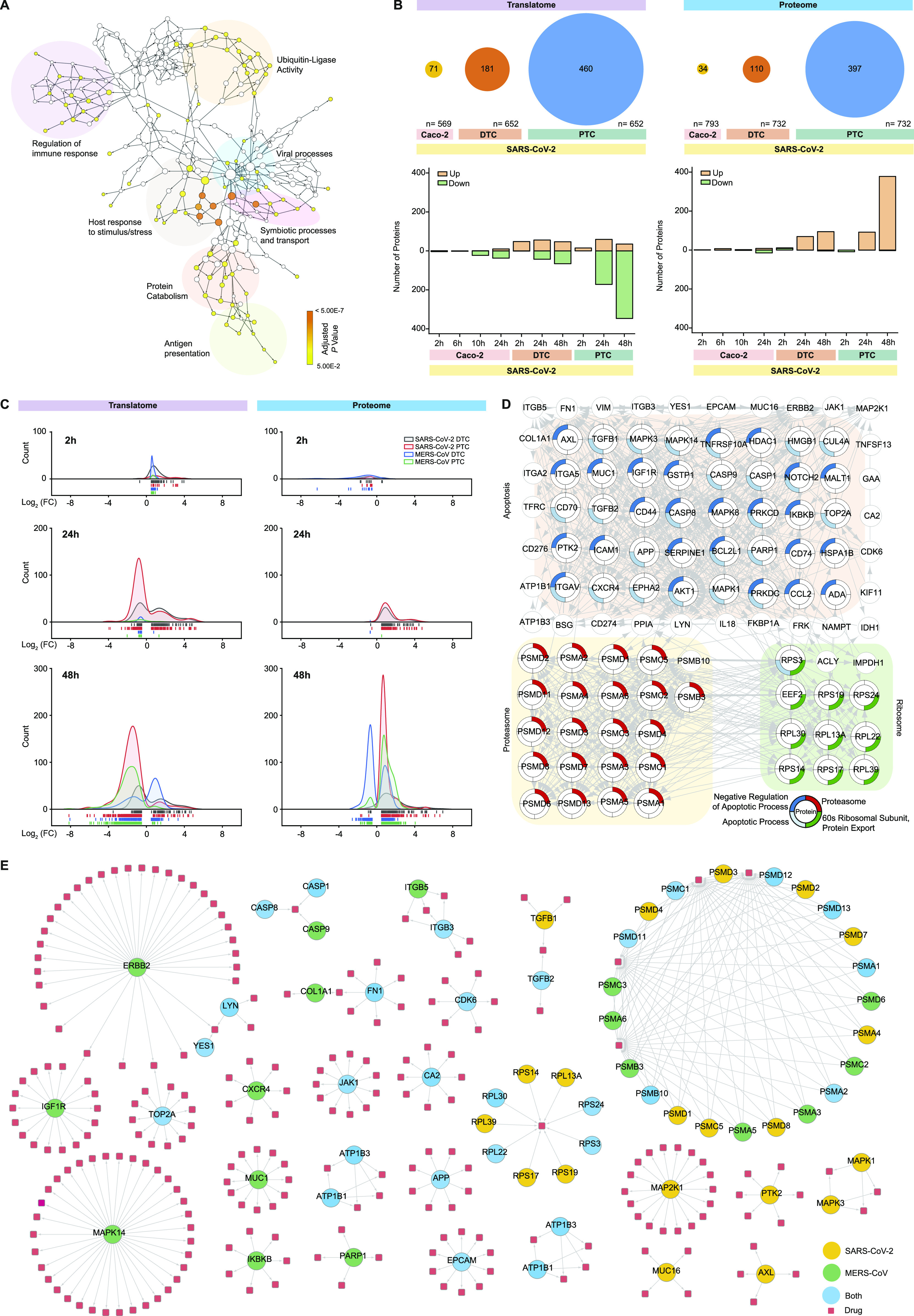
Immune profile changes and corresponding drug targets for SARS-CoV-2 and MERS-CoV infection. **(A)** Biological process enrichment analysis for 75 proteins that mimic viral translation in both PTC and DTC upon SARS-CoV-2 infection. BiNGO Cytoscape plugin was used to create enrichment network. Major pathway sub-groups are highlighted. Color bar represents adjusted *P*-value for BiNGO enrichment. **(B)** SARS-CoV-2 immune response profile in renal epithelial cells compared with Caco-2 cell line (32). Immune system process (GO: 0002376, Proteins in subset = 3,315) GO-term was used for the comparison. The total number of quantified proteins (n) across the conditions are indicated along with number of significantly changing proteins given inside the circles and corresponding histograms showing directionality of change - “up”: increased abundance and “down”: decreased abundance. **(C)** Temporal distribution profiles of significantly changed immune system process (GO: 0002376) proteins in response to SARS-CoV-2 or MERS-CoV infection in PTC and DTC. Data are represented with a quantitative distribution of the mean log_2_ fold-changes (FC). **(C, D)** Proteins from the 48 hpi subsets (from (C)) that had a corresponding drug target in the ChEMBL database. Proteins were locally clustered in three sub-groups with four selected STRING enrichments (STRING plugin for Cytoscape). **(D, E)** Protein–drug network based on (D). Selected data for most interesting drug candidates and corresponding protein targets are shown (complete network and drug target list in Fig S6). Red squares indicate small molecular compound drug candidates available for shown target proteins. Significance cutoff: −0.5 ≥ log_2_ fold-change ≥ 0.5, *P*-value ≤ 0.05.

Table S2 List of top 100 host proteins following viral translation upon infection of PTC and DTC with either SARS-CoV-2 or MERS-CoV (Figs 3A and B and 4A).

**Figure S4.**
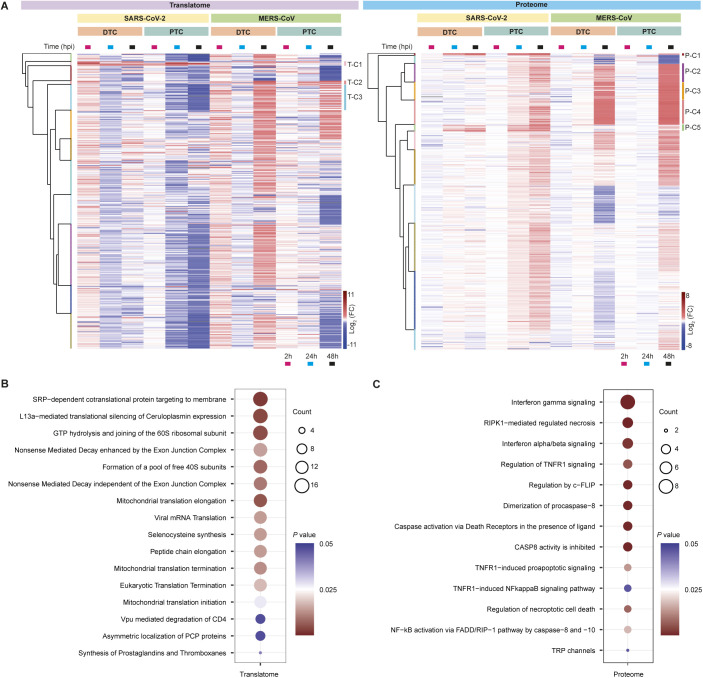
Protein clustering and pathway enrichment analyses for SARS-CoV-2 or MERS-CoV–infected cells. **(A)** Complete hierarchical clustering overview (for Fig 3D) of proteins detected in both viral infections. Each row represents the mean log_2_ fold-change (FC) for a protein upon viral infection compared with control. Total number of proteins: 4,602 in translatome and 5,245 in proteome. **(B, C)** Pathway enrichment analyses using DAVID web-tool (Table S3). Pathways with *P*-value ≤ 0.05 are shown. **(B)** Reactome pathway analysis for cluster 3 (T-C3) of the translatome. Proteins in this subset are increased upon MERS-CoV infection. **(C)** Reactome pathway analysis for cluster 5 (P-C5) of the proteome. This cluster contains proteins increased upon SARS-CoV-2 infection.

**Figure S5.**
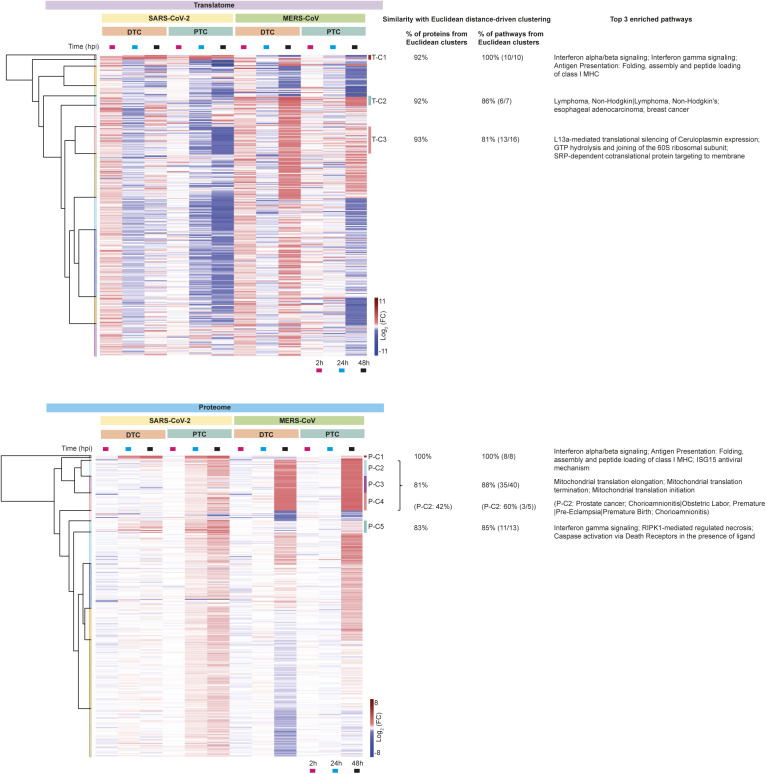
Hierarchical clustering and pathway enrichment validation using Manhattan distance. Complete hierarchical clustering overview of proteins detected in both viral infections using Manhattan distance to validate the Euclidean distance-driven clustering in Fig S4. Each row represents the mean log_2_ fold-change (FC) for a protein upon viral infection compared to control. Total number of proteins: 4,602 in translatome and 5,245 in proteome. Similarity of proteins and pathways reports the percentage of proteins and significantly enriched pathways found in the Euclidean clusters (Fig S4) to also be present in the Manhattan clusters. The top 3 pathways (reactome pathway enrichment was used for all clusters except T-C2 and P-C2 for which GAD disease enrichment was used) are also reported for the Manhattan clusters.

To gain a better overall understanding of host cell responses across viruses and cell types, we next assessed the translatome and proteome for the 4,602 and 5,245 proteins, respectively, that were quantified across all experimental conditions (Fig S4A). Hierarchical clustering revealed several distinct clusters that were modulated upon infection and showed interesting pathway enrichments (Fig 3D and Table S3): Translatome-Cluster 1 (T-C1) and Proteome Cluster 1 (P-C1) consisted of proteins that were increased in translation and protein abundance in PTC and DTC upon SARS-CoV-2 infection (Fig 3D). Reactome pathway analyses revealed that T-C1 and P-C1 proteins were strongly enriched for immune response, including IFN signaling, antigen presentation and interferon-stimulated gene 15 (ISG15)–mediated antiviral mechanisms (Fig 3E). Cluster 2 (T-C2 and P-C2) were composed of proteins showing enrichment for disease terms with conditions related to kidney failure and cancer (Fig 3F). Cluster 3 (T-C3) from the translatome subset contained proteins with decreased translation after SARS-CoV-2 infection and was enriched for proteins involved in translation and 40 and 60 s ribosomal subunits (Fig S4B). The proteome data additionally revealed similarly behaving clusters (P-C2, P-C3, and P-C4) that were defined by extensive increase in host protein levels, particularly at 48 hpi and were strongly enriched for various mitochondrial functions (Fig 3G). Changes in apoptosis and necroptosis pathways were particularly observed at the proteome level upon SARS-CoV-2 infection (Fig 3D P-C5, Fig S4C). Manhattan distance for hierarchical clustering validated these Euclidean distance-driven protein clusters and pathway enrichments, confirming consistent and substantial overlap of our findings (Fig S5). Together, these analyses suggested substantial host cell remodeling that differed between cell types and viruses.

Table S3 Results of pathway enrichment analyses of protein clusters of interest (Figs 3D–G and S4B and C) using DAVID bioinformatics web-tool.

### Primary cells respond to SARS-CoV-2 infection by changing the global immune response profile

We next focused on the host cell responses elicited by SARS-CoV-2. First, we evaluated the set of 75 proteins, which we had identified to follow the viral profile in SARS-CoV-2–infected PTC and DTC (Fig 3B), for specifically enriched biological processes (Fig 4A and Table S4). These analyses revealed distinct clusters and pathways, such as the regulation of immune responses, viral processes, host response to stimulus/stress and antigen presentation as well as ubiquitin-ligase activity, which are commonly known to be part of a global and interconnected immune response to infection (36, 37). Previous proteomic studies for SARS-CoV-2 predominantly used immortalized cell lines, which often induce only limited immune responses. We next focused on the specific immune response signature generated upon infection of the primary renal tubular epithelial cells. To gain further insights into the temporal control of the immune response network, we analyzed all quantified host proteins part of the gene ontology (GO) term – “Immune system process” that changed significantly (−0.5 ≥ log_2_ fold-change ≥ 0.5, *P*-value ≤ 0.05) for the different time points, cells and viruses (Fig 4B and C). First, we compared the extent of immune response upon SARS-CoV-2 infection between previously studied Caco-2 cells (32) and the primary renal tubular epithelial cells in this study (Fig 4B). Using the immune system process dataset, we quantified approximately the same number of proteins for both Caco-2 and primary renal tubular epithelial cells. However, despite the large difference in MOI used in the different studies (MOI of 1 for Caco-2 cells versus MOI of 0.01 for primary renal epithelial cells), the total number of significantly changed proteins varied largely between cell types, with a stronger immune response observed in the primary renal epithelial cells (Fig 4B). The directionality of change and the extent of response in PTC in principle make this a good model system for studying changes in the host immune profile upon SARS-CoV-2 infection.

Table S4 Results of gene ontology analysis of the 75 host proteins that followed the SARS-CoV-2 viral profile in both PTC and DTC (Fig 4A).

Next, we compared immune profile changes across viruses corresponding to the GO-term “Immune system process” (Fig 4C). MERS-CoV elicited robust immune profile changes at 48 hpi. Contrary to this observation, SARS-CoV-2 infection led to earlier and more complex immune responses. Here, a greater number of immune proteins were significantly changed at 24 hpi in both PTC and DTC, with effects in the translatome being more pronounced than in the proteome. This finding is consistent with the higher time-resolution of translatome measurements. Whereas translatome changes were distributed in host proteins with both increased and decreased translation, our proteome measurements showed only an increase in immune protein levels. The translatome of SARS-CoV-2–infected cells at 48 hpi showed a further shift of immune proteins to significantly reduced translation, consistent with attempts to shut down the antiviral host response. These effects were more pronounced in PTC (347 of 382 proteins) versus DTC (66 of 113 proteins). However, immune protein levels further increased in SARS-CoV-2–infected PTC (378 of 381 proteins) at 48 hpi, which we did not observe in DTC (94 of 99 proteins). Cells infected with MERS-CoV exhibited a delayed immune response profile with pronounced translatome effects at 48 hpi, reflecting the observations made for SARS-CoV-2 cells at 24 hpi, despite comparable levels of viral proteins, for which no delay was observed across viruses (Fig 2D and E). MERS-CoV–infected PTC largely showed decreased translation of immune proteins (206 of 213 changed proteins), whereas DTC had similar numbers of immune proteins translationally increased or decreased (114 and 79, respectively). Immune proteome levels of MERS-CoV–infected cells showed a large fraction of increased proteins in PTC (322 of 357 changed proteins) at 48 hpi, reminiscent of SARS-CoV-2–infected PTC at 48 hpi. In DTC, similar fractions of proteins were increased or decreased in abundance (147 and 222 proteins, respectively). Overall, our data highlight that SARS-CoV-2–infected cells showed an earlier and more severe immune response with extensive differences between PTC and DTC, offering a potential explanation for the observed differences in CPE development and viral titers between SARS-CoV-2 and MERS-CoV.

**Figure S6.**
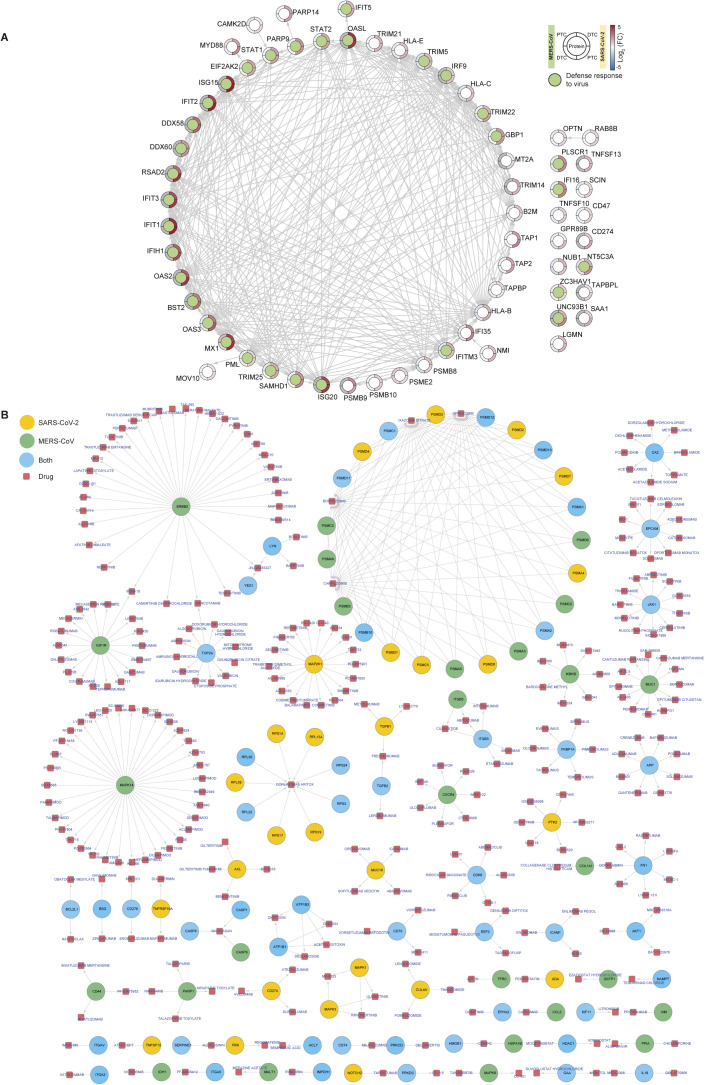
Immune response specific drug network analysis for SARS-CoV-2 and MERS-CoV. **(A)** Proteins with significantly increased translation in either SARS-CoV-2– or MERS-CoV–infected samples at 24 hpi (Fig 4C, translatome) that correspond to the GO-term Immune system process (GO: 0002376) are shown as a STRING network. A large subset of these proteins belonged to the GO-term “Defense response to virus” (GO: 0098542, indicated in green) upon STRING functional enrichment (STRING plugin for Cytoscape). Outer ring shows mean log_2_ fold-change (FC; colorbar) for protein in infected samples compared with controls for each viral infection and cell type. Significance cutoff: −0.5 ≥ log_2_ fold-change ≥ 0.5, *P*-value ≤ 0.05. **(B)** Complete protein–drug network (for Fig 4E). Proteins that belonged to GO-term Immune system process (GO: 0002376) and were significantly changed at 48 hpi in response to infection are shown alongside their corresponding small molecular compound drug candidates.

To further identify early drivers of this immune response, we focused on the 64 immune response proteins that were significantly increased at 24 hpi in any of the four subsets (Fig S6A). MX1 was the only protein that was increased in all groups and the only immune response protein to be increased in response to MERS-CoV infection at 24 hpi. SARS-CoV-2–infected cells showed significant changes for 51 of the 64 proteins. Many of these host proteins were associated with IFN signaling, defense responses and negative regulation of viral processes. Interestingly, we observed opposite effects on the human leukocyte antigen system or MHC-class I proteins human leukocyte antigen-B/C/E, which were increased for SARS-CoV-2–infected and decreased for MERS-CoV–infected cells. Alongside transporters associated with antigen presentation 1 and 2 (TAP1 and TAP2) proteins, we also observed a SARS-CoV-2–specific increase of oligoadenylate synthase (OAS) antiviral response proteins (OAS2/3/L and DDX58, log_2_ fold-change >2.5), which impose the IFN-induced antiviral activity on replication of coronaviruses (38).

To identify immune response targets, for which small molecule drugs are available, we combined proteins that were significantly altered in translatome and/or proteome at 48 hpi and integrated these with the ChEMBL database. Using this approach, we identified 104 druggable target proteins. STRING enrichment analysis of these targets identified three major pathways: regulators of apoptosis, proteasomal subunits, and proteins associated with processing of the 60 s ribosomal subunit (Fig 4D). We created a network of these 104 targets and more than 300 potentially therapeutic drug candidates (Figs 4E and S6B). Among these, we identified the drugs ribavirin, bortezomib, and gilteritinib, which have also been identified as potential therapeutic candidates in previous SARS-CoV-2 studies (39, 40). In addition, we found fibronectin 1 (FN1) to be significantly changed in both MERS-CoV and SARS-CoV-2–infected cells, exhibiting a similar pattern at the translatome (log_2_ fold-change ≤ −0.5, *P*-value ≤ 0.05) and proteome level (log_2_ fold-change ≥ 0.5, *P*-value ≤ 0.05; except SARS-CoV-2–infected DTC). We also identified DNA Topoisomerase II Alpha (TOP2A) and its corresponding inhibitor mitoxantrone, a drug relevant for both SARS-CoV and SARS-CoV-2 (41). In SARS-CoV-2–infected PTC, TOP2A translation was significantly decreased at 48 hpi, whereas DTC showed a significant increase in TOP2A levels for both translatome and proteome in response to MERS-CoV infection.

**Figure S7.**
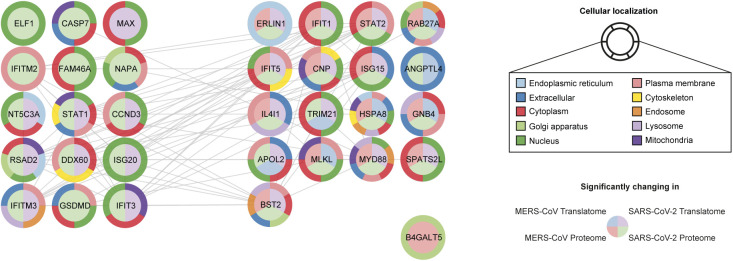
Network of significantly changing SARS-CoV-2 inhibitory ISGs in response to SARS-CoV-2 or MERS-CoV infection. SARS-CoV-2 inhibitory ISGs significantly changing at any time post-infection in the translatome or proteome in response to SARS-CoV-2 or MERS-CoV infection of either PTC or DTC are shown. Proteins are clustered with respect to their relevance for either SARS-CoV-2 or MERS-CoV infection or both. Cellular localization was determined by COMPARTMENTS subcellular localization database in conjunction with GeneCards database. Network was generated using STRING plugin for Cytoscape. SARS-CoV-2 inhibitory ISG dataset (65 proteins) was obtained from Martin-Sancho et al (2021) (42). Significance cutoff: −0.5 ≥ log_2_ fold-change ≥ 0.5, *P*-value ≤ 0.05. Only proteins significantly changed in our dataset are shown (33 proteins).

IFN response and interferon-stimulated genes (ISGs) appear to play an important role in the antiviral SARS-CoV-2 host response. Recently, 65 ISGs were reported to inhibit SARS-CoV-2 replication (42). We next evaluated our results in comparison with these described ISGs and found them to correlate. We quantified 32 and 27 of these ISGs in our SARS-CoV-2 and MERS-CoV dataset, respectively (Fig S7 and Table S5). Of these, we identified 32 and 18 ISGs to be significantly changed in at least one cell type for one time point after infection with SARS-CoV-2 or MERS-CoV, respectively. This high level of overlap emphasizes the important role of ISGs across different cells and coronaviruses.

Table S5 SARS-CoV-2 inhibitory ISGs reported by Martin-Sancho et al (2021) (42) were compared with all the quantified ISGs (from this list) in our dataset to identify significantly changing ISGs in response to SARS-CoV-2 and MERS-CoV infection in primary renal epithelial cells (Fig S7).

### SARS-CoV-2 and MERS-CoV alter the mitochondrial protein landscape

**Figure 5.**
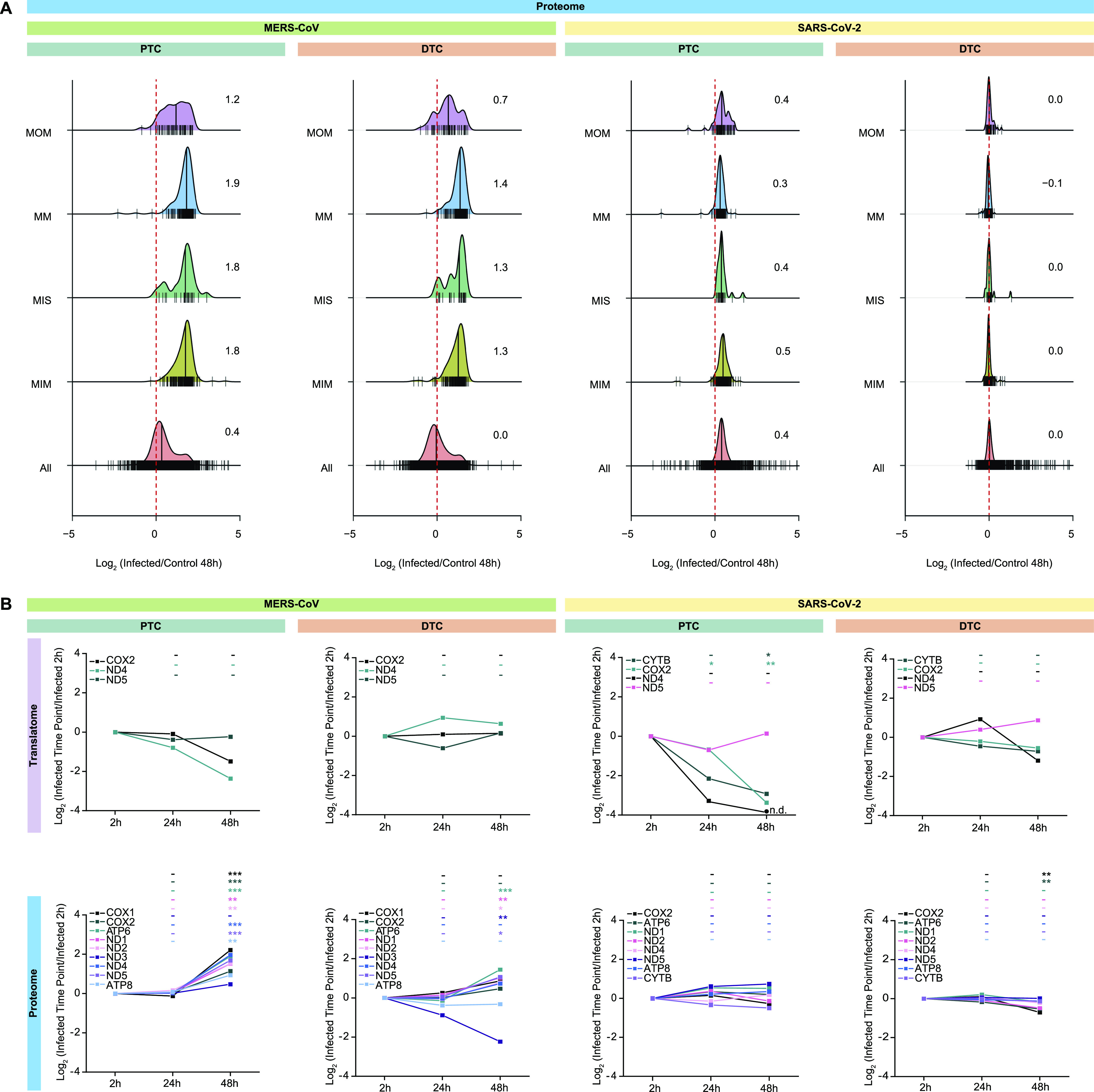
SARS-CoV-2 and MERS-CoV differently affect mitochondrial protein dynamics. **(A)** Density plots showing distribution of the mean log_2_ fold-changes of all quantified host proteins compared with mitochondrial proteins belonging to different suborganellar locations. Numbers and ridges represent median and protein count, respectively. Suborganellar localization of mitochondrial proteins was obtained from MitoCarta2.0. MOM, mitochondrial outer membrane; MM, mitochondrial matrix; MIS, mitochondrial intermembrane space; MIM, mitochondrial inner membrane; All, all quantified host proteins. **(B)** Abundance and translation changes of mitochondrial-encoded proteins. Shown are the mean log_2_ fold-changes at different times post-infection compared with the corresponding 2 hpi samples (n = 3 independent biological replicates). *t* test significance is indicated as ***P-value < 0.001; **P-value < 0.01; *P-value < 0.05; -: not significant.

**Figure S8.**
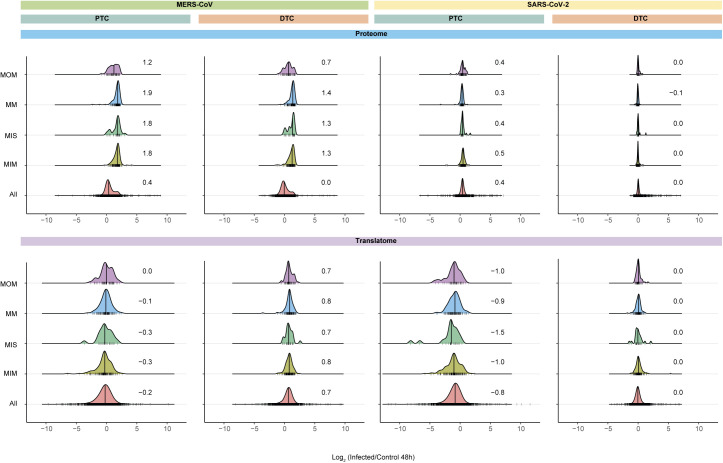
Mitochondrial proteome and translatome changes in response to SARS-CoV-2 or MERS-CoV infection. Density plots showing distribution of mean log_2_ fold-changes of all quantified newly synthesized host proteins compared with mitochondrial proteins belonging to different suborganellar compartments, upon viral infection compared with controls. Data are shown for proteome (complete range, for Fig 5A) and translatome at 48 hpi. Numbers and ridges represent median and protein count, respectively. MOM, mitochondrial outer membrane; MM, mitochondrial matrix; MIS, mitochondrial intermembrane space; MIM, mitochondrial inner membrane; All, all host proteins.

Viruses often depend on mitochondria to evade the host immune response (43). Thus, we next studied the effects of SARS-CoV-2 or MERS-CoV infection on mitochondrial dynamics within submitochondrial compartments. We found a predominant increase in mitochondrial proteins across all submitochondrial compartments upon infection with MERS-CoV in both PTC and DTC at 48 hpi (Fig 5A). This effect was not due to overall changes in the cellular protein levels, as the global proteome remained largely unchanged. Strikingly, this effect was not observed in SARS-CoV-2–infected cells, which did not exhibit different patterns when compared with the total proteome (Fig 5A). To address whether changes in the 13 proteins encoded by the mitochondrial genome are predominantly driven by extra-mitochondrial factors, we monitored synthesis and abundance changes of the quantified mitochondrial-encoded proteins (Fig 5B). Although we observed only minor differences in SARS-CoV-2–infected cells, mitochondrial-encoded proteins overall increased upon infection with MERS-CoV, consistent with findings in nuclear-encoded mitochondrial proteins (Fig 5A and B). In accordance with translation rates of mitochondrial proteins that did not change upon infection (Fig S8), these findings suggest an increase in mitochondrial mass. Overall, these findings may explain the accelerated immune response to SARS-CoV-2 versus MERS-CoV–infected cells, potentially driven by altering mitochondrial function (44).

### Mitochondrial and nuclear pore alterations potentially contribute to renal pathology over an inflammatory background

Severe cases of infection with SARS-CoV-2 or MERS-CoV can result in renal pathology (26, 45). Kidney diseases are increasingly associated with mitochondrial dysfunction and inflammation (46, 47), key features of the effects we observed upon infection (Figs 4 and 5). Particularly, renal tubular cells are highly dependent on energy for a multitude of transport processes. Thus, changes in the mitochondrial system are one of the main reasons for AKI (48, 49, 50).

**Figure 6.**
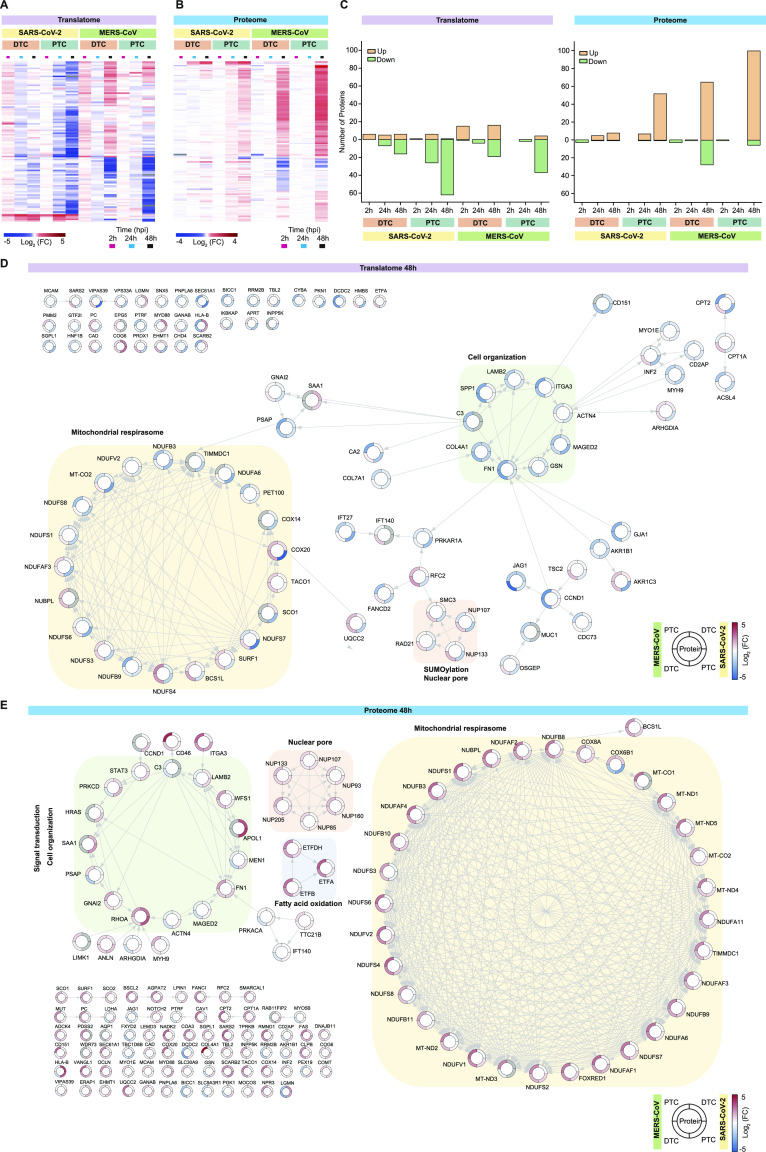
Renal pathology-related changes upon SARS-CoV-2 or MERS-CoV infection. **(A, B)** Hierarchical clustering of proteins associated with renal function and pathology in translatome (A) and proteome (B) upon SARS-CoV-2 or MERS-CoV infection. Each row represents the mean log_2_ fold-change (FC) for a protein upon infection compared with control. Proteins significantly changed in any one subset are shown. GSEA datasets used are acute kidney injury (HP:0001919), chronic kidney disease (HP:0012622), renal insufficiency (HP:0000083), GO-term renal system process (GO:0003014), renal tubular dysfunction (HP:0000124), and renal tubular atrophy (HP:0000092). **(C)** Quantitative analysis of significantly increased or decreased proteins (from A and B). **(D, E)** STRING network for significantly changed proteins (in any one subset) at 48 hpi in the translatome (D) and proteome (E). Color bar in the outer ring represents the mean log_2_ fold-change for proteins upon infection compared with controls. Functionally related protein clusters are highlighted. Significance cutoff: −0.5 ≥ log_2_ fold-change ≥ 0.5, *P*-value ≤ 0.05.

To better understand the effects of SARS-CoV-2 or MERS-CoV infection in respect to the molecular mechanisms underlying renal pathology, we studied proteins that are part of the GSEA datasets – “AKI,” “chronic kidney disease,” “renal insufficiency,” “renal system process,” “renal tubular dysfunction,” and “renal tubular atrophy.” We identified 104 (of possible 115, translatome) and 130 (of possible 143, proteome) quantified proteins for both viruses that significantly (−0.5 ≥ log_2_ fold-change ≥ 0.5, *P*-value ≤ 0.05) changed in any one subset. Hierarchical clustering of these proteins showed major virus-specific changes in the translatome (Fig 6A) and to a lesser extent in the proteome (Fig 6B). Similar to the observations obtained globally (Fig S4A), translation of kidney disease related proteins was largely decreased, especially upon SARS-CoV-2 infection. However, we identified distinct protein clusters that showed virus-specific differences. Overall, the decrease in translation of kidney disease associated proteins was more pronounced in PTC when compared with DTC (Fig 6A). In the kidney disease–related proteome, changes across cell types were more extensive than virus-driven effects, with more extensive proteome increases in PTC (Fig 6B).

Next, we examined the specific subset of proteins that changed significantly (−0.5 ≥ log_2_ fold-change ≥ 0.5, *P*-value ≤ 0.05; Fig 6C). We observed pronounced cell type effects with PTC showing translational suppression. DTC exhibited an even distribution of proteins that translationally increased or decreased. However, DTC showed differences in response to the individual coronaviruses. Whereas SARS-CoV-2 infection resulted in modest changes, MERS-CoV infection yielded a robust response comparable with PTC at 48 hpi.

**Figure S9.**
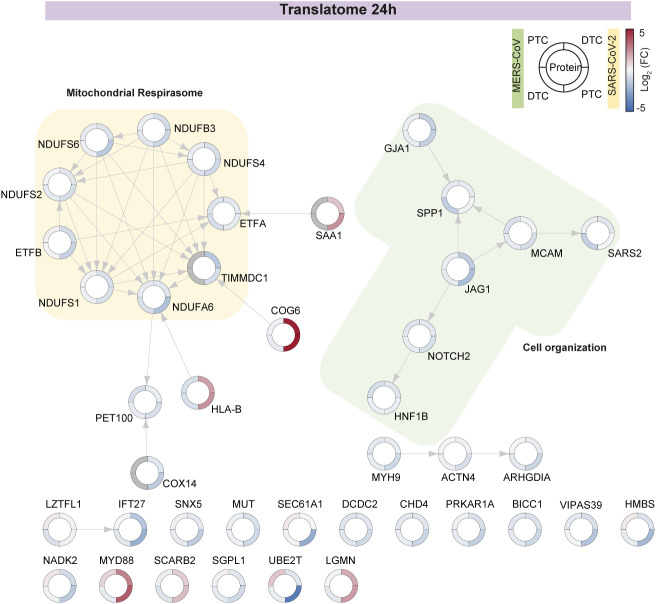
Early changes in translation of proteins relevant for renal pathology. STRING network of proteins with significantly changed translation (See Fig 6A) in any one subset at 24 hpi. Colorbar in the outer ring represents mean log_2_ fold-change (FC) for protein levels upon infection compared with controls (n = 3 biological replicates). Functionally related protein clusters are highlighted and indicated. Significance cutoff: −0.5 ≥ log_2_ fold-change ≥ 0.5, *P*-value ≤ 0.05.

To identify early changes, we focused on 41 proteins that were significantly changed in any one subset at 24 hpi (Fig S9). Interestingly, we observed translational attenuation of mitochondrial complex I components and Notch signaling. At 48 hpi, effects on mitochondria were more predominant, along with changes in cell organization at the level of a few endoplasmic reticulum proteins (Fig 6D). Among the subnetworks, we identified respiratory electron transport, SUMOylation and extracellular matrix. At the translatome level, proteins that predominantly increased upon MERS-CoV infection in DTC were part of the respirasome as well as SUMO and nuclear pore clusters. This observation corresponded with proteome changes in MERS-CoV–infected cells (Fig 6E). Interestingly, SARS-CoV-2–infected PTC had a similar, although weaker, response. Overall, the proteome changes at 48 hpi revealed clusters such as the tricarboxylic acid cycle, endocytosis and transport, nuclear pore formation and function, and peroxisome/ketone metabolism, all of which may play a role in viral infection. In particular, nuclear pore protein NUP93 was shown to bind the SARS-CoV Nsp1 protein, a potential mechanism of host translation modification by the virus (51). Consistent with previous observations from patients with renal pathology, we also observed FN1 protein accumulation, decreased complex I activity and immune activation (46, 52). Therefore, our primary renal tubular epithelial cell infection system serves as a powerful model to study immune responses of the host cell as well as the potential pathways resulting in the corresponding renal pathological effects. These data may enable us to analyze suitable drugs that have the potential to prevent coronavirus-mediated acute renal failure in the future.

## Discussion

The impact of highly pathogenic coronaviruses including SARS-CoV, MERS-CoV, and recently SARS-CoV-2 on renal pathology has been largely documented for critically ill patients (18, 53). Recent clinical reports suggested a high prevalence of AKI in hospitalized cases of COVID-19 (30–78%) associated with increased mortality rates (5, 6, 45, 54, 55). The molecular pathogenic mechanisms of COVID-19–associated AKI are diverse and currently not clearly understood. Acute tubular epithelial cell injury, systemic inflammatory response induced by a cytokine storm, and endothelial dysfunction appear to be the contributing mechanisms of AKI (56). Furthermore, renal histopathological analysis identified coronavirus-like particles with distinctive spikes in the cytoplasm of the proximal and also, but less so, in more distal tubules (17). MERS-CoV’s renal tropism along with efficient viral infection has previously been observed in primary human kidney cells and is distinctive in its severity and prominence in the kidney from other coronaviruses (20). Despite these clinical and experimental observations, the global host cell responses to such viral infections in the kidney have remained poorly understood. Here, we used global whole-cell proteomics to compare host cell responses induced by MERS-CoV and the novel coronavirus SARS-CoV-2 in highly purified primary renal proximal and distal tubular epithelial cells (27). We here provide with the first assessment of proteome and translatome changes in primary renal cells for SARS-CoV-2 and any cell type for MERS-CoV, allowing high temporal resolution of infection effects.

In agreement with earlier findings on renal epithelial cells (20), MERS-CoV showed successful infection with linear growth in viral replication accompanied by the development of CPE in both PTC and DTC. Compared to MERS-CoV, we observed delays in the onset of CPE induced by SARS-CoV-2, correlating with lower viral titers at 48 hpi. Unlike suggested by previous findings in COVID-19 patients (17, 57), we found no clear preference for PTC over DTC in regard to viral replication. A preference for PTC has been explained by higher expression levels of ACE-2, the primary receptor for SARS-CoV-2 entry, present predominantly in the proximal tubules (57, 58). However, transmembrane serine protease 2 (TMPRSS2), cathepsin L, and furin proteases required for SARS-CoV-2 spike protein cleavage and viral entry are also abundantly expressed in DTC (59, 60, 61). Interestingly, recent studies show that DTC instead of PTC may be the primary target of SARS-CoV-2 induced AKI and are positive for SARS-CoV-2 viral RNA along with ACE-2 and TMPRSS2 (62, 63). Therefore, efficient SARS-CoV-2 entry may rely on multiple host factors or modes of viral uptake in these cells.

We observed similarities as well as differences in global host cell responses across PTC and DTC infected with SARS-CoV-2 or MERS-CoV, indicating cell-specific and shared responses. We found PTC to be generally more susceptible to infection-associated host cell remodeling, in particular for SARS-CoV-2 infection exhibiting severe translational attenuation at 48 hpi. This may be explained by studies showing that SARS-CoV-2 protein Nsp1 inhibits host cell translation, mediated by its binding to the 40s ribosomal subunit (64, 65, 66). Consistently, we observed negative enrichment for reactome pathways “GTP hydrolysis and joining of the 60s subunit” as well as “formation of a pool of free 40s subunits.” In contrast, MERS-CoV infection in both PTC and DTC resulted in a positive enrichment for these pathways, probably due to the nuclear localization of MERS-CoV Nsp1 and its unstable binding properties with the 40 s subunit, unlike in SARS-CoV-2 (67). Overall, PTC were more susceptible to translational inhibition than DTC. Interestingly, this effect was stronger upon SARS-CoV-2 infection in our study despite previous evidence supporting efficient translation inhibition by MERS-CoV Nsp1, albeit through other mechanisms (67, 68).

Strikingly, we observed a significant difference in immune response of primary renal tubular cells to SARS-CoV-2 or MERS-CoV infection. MERS-CoV uses different strategies to inhibit the activation of the IFN response and effectively evades the host immune system (69, 70). We observed a similar effect upon infection that resulted in delayed host cell responses in both the translatome and proteome. However, SARS-CoV-2 exclusively activated some early immune response proteins that mimicked the viral translation profile at 24 hpi. Among these immune responses, we observed the activation of various pathways, such as IFN signaling, ISGylation, proteasomal system and the endoplasmic reticulum–phagosomal axis. We also observed one cluster containing proteins with increased abundance upon SARS-CoV-2 infection that corresponded to differential regulation of necrosis and apoptotic signaling pathways, which may explain the differences in CPE in response to different viruses.

The strength of our system is the extensive immune activation that we observe upon infection of the primary cells. Furthermore, primary renal epithelial cells are particularly useful to study coronavirus-associated renal pathology. Our translatome data highlighted early changes in immune response proteins after SARS-CoV-2 infection, which, similar to the global protein profile, succumbed to translational attenuation effects at 48 hpi, particularly in PTC. In contrast, the proteomic profile for the latter continued to increase and could explain the cytokine storm associated with SARS-CoV-2 infection and the pro-inflammatory response-driven changes in kidney pathology (53, 71, 72, 73, 74). MERS-CoV infection yielded a similar response although DTC showed a more bidirectional effect. Focusing on this axis, we identified more than 100 immune targets altered by either one or both viral infections. Their corresponding 300 drug candidates may potentially contribute to a specific or multi-faceted approach for the treatment of renal pathology associated with highly pathogenic coronaviruses. We found that these targets were largely ribosomal proteins (60 s subunit), apoptotic regulators and proteasomal subunits. We identified various drug candidates such as cyclosporine and alisporivir targeting cyclosporine A, previously shown to inhibit MERS-CoV replication (30), ribavirin which may be effective against both SARS-CoV-2 and MERS-CoV as well as bortezomib, gilteritinib, and mitoxantrone, which have been reported for their potential therapeutic effects against COVID-19 (39, 40, 41, 75).

Although future studies will be required to elucidate the factors involved in activating pathways driving coronavirus-associated kidney damage, our study provides with key observations suggesting possible mechanisms. Renal function and disease pathology proteins altered in response to infection highlighted mitochondrial, nuclear pore and cell organization proteins. Protein abundance of mitochondrial respirasome and nuclear pore complex components predominantly increased in response to MERS-CoV infection as well as in SARS-CoV-2–infected PTC. At the proteome level, we observed distinctive similarity in a subset of proteins that were increased in PTC infected with SARS-CoV-2 and in general, upon MERS-CoV infection. This subset included proteins enriched for diseases such as kidney aging, renal failure, type-2 diabetes and numerous cancers as well as various mitochondrial pathways. Mitochondria play an important role in AKI and host immune responses and previous studies have shown mitochondrial hijacking by coronaviruses for efficient replication and immune evasion (76, 77, 78). SARS-CoV-2 protein interaction maps showed the affiliation of viral proteins with different mitochondrial proteins as well as mitochondrial localization signals (79, 80). In SARS-CoV-2–infected PTC, we observed a significant reduction in the translation of the mitochondrial import receptor subunit TOM70 which was recently reported to interact with SARS-CoV-2’s ORF9b and exhibited decreased expression upon SARS-CoV-2 infection in Caco-2 cells (79). We also observed a reduction in translation of mitochondrial complex I proteins, consistent with previous findings (81). In our study, MERS-CoV resulted in an altered mitochondrial profile, with increased protein levels of both nuclear- and mitochondrial-encoded proteins, an effect not observed for SARS-CoV-2. This difference may correspond to the difference in immune activation versus evasion observed for the two phylogenetically distinct coronaviruses.

Apart from globally enriched pathways, we identified a few interesting proteins which may be subject of future studies. This includes MX1, the only antiviral effector and immune response protein to be activated by both SARS-CoV-2 and MERS-CoV at 24 hpi. *MX1* expression levels were reported to be significantly increased in COVID-19 patients (35). Notably, *MX1* can be induced by hemin, a United States Food and Drug Administration approved drug, which exhibits inhibitory effects on SARS-CoV-2 replication (35, 82 *Preprint*). We also found FN1 levels to be similarly changed by both viruses and therefore representing a potential shared therapeutic target that not only plays a role in immune response and cell organization but also in kidney pathology. Previously, human fibronectin protein-based intrabodies were shown to inhibit SARS-CoV replication by targeting the nucleocapsid protein (83). TOP2A inhibitor mitoxantrone identified in our study was recently shown to inhibit SARS-CoV and SARS-CoV-2 viral entry and could also have beneficial effects in preventing SARS-CoV-2 infection of the kidney (41). Nuclear pore complex proteins are also targets of various viral infections and NUP93 has specifically been shown to be an important player in viral mRNA nuclear-cytoplasmic export (84, 85, 86). Early drivers of SARS-CoV-2 IFN response including MHC-class I, TAP, and OAS antiviral response proteins are also interesting candidates.

Our study demonstrates the strength of using primary cells to study host cell immune responses to viral infection and offers a glimpse into the balance between virus- and host-generated responses that alter translation and influence global protein accumulation. Our primary epithelial cell culture model further highlights the multi-etiological origins of coronavirus-associated renal pathology–mitochondrial rearrangements as well as immune responses in particular. We observed a stronger impact on renal proximal tubular epithelial cells, which is consistent with clinical findings for SARS-CoV-2 infection (17, 57). Finally, our data provide insights into potential molecular mechanisms underlying renal pathogenesis associated with coronavirus infections which may aid in the development of future anti-coronavirus therapies.

### Limitations

The primary cells used were obtained in limited amounts as surplus material from surgery. Consequently, they have to be combined with highly sensitive methods that are capable of quantifying small numbers of cells, limiting the number of possible assays. Furthermore, the primary PTC and DTC used here are well differentiated and therefore, highly reflect these nephronal cells in vivo, providing an improved insight into immune responses when compared with immortalized cell lines (Fig 4B). However, they do not reflect the complexity of the in vivo situation, particularly in regard to the spatial environment in the tissue. Thus, although our infection system provides a relevant in vitro model to assess renal cell host responses, it cannot recapitulate the breadth of pathological effects underlying AKI in COVID-19 patients. Therefore, our findings, particularly in the context of possible molecular targets, will require further evaluation in in vivo models for SARS-CoV-2 infection.

## Materials and Methods

The study was conducted according to the guidelines of the Declaration of Helsinki and examined by the Ethics Committee of Clinics of the Goethe-University. Because of the complete anonymization of all patient data, an ethics vote was waived by the local ethics committee.

### Informed consent statement

Informed consent was obtained from patients involved in the study.

### Isolation, culture, and characterization of primary epithelial cells

Human renal proximal and distal tubular epithelial cells were isolated using antibody-coated magnetic beads as described previously (27, 29). In brief, cells were prepared after tumor nephrectomies from portions of the kidney not involved in renal cell carcinoma. First, the tissue was minced and digested with collagenase/dispase. Then, the digested fragments were passed through a 106 μm mesh and incubated with collagenase IV, DNase, and MgCl_2_. After Percoll density gradient centrifugation, unspecific binding sites were blocked by preincubation with human immunoglobulin G (hIgG, 5 mg/ml). To enrich PTC, an antibody against aminopeptidase M (APM, ANPEP, and CD13) was used. DTC were isolated using an antibody recognizing Tamm-Horsfall glycoprotein (Uromucoid), a specific antigen of the thick ascending limb of Henle’s loop and the early distal convoluted tubule. Finally, cells were incubated with a bead-conjugated secondary antibody and isolated by immunomagnetic separation applying the Mini-MACS system (Miltenyi). Isolated cells were seeded in six-well plates precoated with FBS and were grown in medium 199 (Sigma-Aldrich) with a physiological glucose content, 10% FBS at 37°C and 5% CO_2_ in a humidified atmosphere. Cells were passaged by trypsination.

Primary isolated and cultured cells were comprehensively characterized by various cell biological methods (27, 29). Primary isolated PTC are strongly positive for aminopeptidase M; however, isolated cells of the distal portion are strictly negative (27). Cultured PTC highly express aquaporin-1 and ICAM-1, whereas E-cadherin is highly expressed in cultured DTC. The formation of a dense epithelial cell monolayer on cell culture plastic was further demonstrated by the formation of microvilli and tight junctions as well as the expression of zonula occludens protein 1 (29). Ultrastructural analysis by scanning electron microscopy revealed long microvilli on the apical surface of PTC, indicating cellular polarity, whereas cultured DTC only develop short microvilli on their apical surface membrane.

### Infection and virus titration

Primary PTC and DTC were grown in chamber slides for immunofluorescence analysis, or 12-well plates for growth kinetics and transcriptome and proteome analyses. Cells were maintained at 37°C in an atmosphere containing 5% CO_2_. Triplicates of each cell line either were infected with SARS-CoV-2 (BavPat1/2020 isolate, European Virus Archive Global # 026V-03883) or MERS-CoV (strain EMC/2012), or were left untreated (non-infected control cells). Based on our previous observation that MERS-CoV caused strong apoptotic cell loss and extensive formation of multinucleated cell foci in infected Calu-3 cells and primary human aortic endothelial cells at 24 h post-infection (hpi) using an MOI of 0.1 (30), PTC and DTC were infected using an MOI of 0.01 to limit the early onset of cytopathic effects and apoptosis in these cells. Supernatants and cell lysates were harvested at 2, 24, and 48 hpi. At 2 h before harvest, cells were washed three times with PBS and transferred to heavy SILAC labeling buffer (84 mg/l L-arginine (^13^C_6_,^15^N_4_ (R10); Cambridge Isotope Laboratories, CNLM-539-H) and 146 mg/l L-lysine (^13^C_6_,^15^N_2_ (K8); Cambridge Isotope Laboratories, CNLM-291-H)). Cells were again washed three times with PBS and lysed in 2% SDS in H_2_O, incubated at 95°C and stored at −80°C until further analysis. Virus titration was performed by defining the 50% tissue culture infectious dose (TCID_50_). For this, the cell culture supernatants were diluted fivefold and used to infect VeroE6 cells (ATCC CRL-1586) in 96-well plates (four wells per dilution). The cultures were scored for cytopathic effects at 5–6 days post-infection. The end point virus titers were calculated using the method of Reed and Muench (87). All infection experiments with SARS-CoV-2 and MERS-CoV were performed under biosafety level 4 biocontainment conditions at the Institute of Virology of the Philipps University Marburg.

### Immunofluorescence microscopy

PTC and DTC were grown in chamber slides and were infected with SARS-CoV-2 or MERS-CoV at an MOI of 0.01. At 24 hpi, cells were washed with PBS and fixed with 4% PFA in DMEM for 48 h at 4°C. After removal of PFA, cells were incubated in DMEM for 1 h. Free aldehydes were quenched with 0.1 M glycine in PBS for 30 min. Then, samples were washed and permeabilized with PBS containing 0.1% Triton X-100 for 30 min at RT. Fixed cells were washed twice with PBS, incubated in blocking solution (0.2% bovine serum albumin in PBS) for 20 min at RT and subsequently stained with the mouse anti-dsRNA J2 monoclonal antibody (Scicons) for 2 h at RT, followed by Alexa Fluor Chicken anti-mouse 488 (Invitrogen) secondary antibody for 1 h at RT. Cell nuclei were stained with DAPI (4′, 6′-diamidino-2-phenylindole; Sigma-Aldrich). After rinsing with PBS, samples were mounted with Fluoroshield. Microscopic analysis was performed using a confocal laser scanning microscope (Leica).

ACE-2 and DPP4 expression levels in PTC and DTC were observed using immunofluorescence staining. In brief, cells were cultured on chamber slides, fixed, and blocked by PBS containing 5% normal goat serum. Primary antibody anti-ACE-2 (UK, No. 15348; final concentration 5 μg/ml; Abcam) was incubated for 30 min at 37°C. After washing with PBS, cells were incubated with a Cy3-conjugated goat–anti-rabbit IgG (UK, No. 111-165-144, 1:300; Jackson ImmunoResearch) for 30 min at 37°C. DPP4 staining was performed using a PE-labeled antibody anti-CD26 (No. 302705, final concentration 2 μg/ml; BioLegend). Nuclei were counterstained with DAPI (blue). Controls of nonspecific fluorescence were performed on fixed cells processed without the primary antibody. Monolayers were mounted in mounting medium and examined using a Keyence BZ-X800 (post-processed with the BZ-X800 analyzer software using haze reduction) or a Zeiss Axiolab fluorescence microscope equipment.

### Flow cytometry

DPP4 expression was additionally assessed in PTC and DTC using a FACSVerse flow cytometer with FACSuite software (BD Biosciences). Cells were labeled using a PE-labeled antibody anti-CD26 (No. 302705; BioLegend). All experiments included corresponding isotype-matched negative controls. Cells were gated by forward and sideward scatter to eliminate cellular debris.

### Sample preparation for LC-MS/MS

SILAC labeled sample lysates in 2% SDS buffer were resuspended in hot lysis buffer (final concentration – 2% SDS, 150 mM NaCl, 50 mM Tris–HCl, pH 8, 10 mM TCEP, 40 mM 2-chloracetamide, and protease inhibitor cocktail tablet [EDTA-free, Roche]). Lysates were incubated for 10 min at 95°C, sonicated for 1 min with 1 s ON/1 s OFF pulse at 30% amplitude using Sonic Vibra Cell, and incubated at 95°C for another 10 min.

Samples were prepared for mass spectrometry as described previously (33). In brief, lysates were methanol-chloroform precipitated and the protein pellets were resuspended using 8 M Urea/10 mM EPPS pH 8.2. Pierce BCA protein assay kit (Thermo Fisher Scientific) was used to determine protein concentration. Samples were diluted to 2 M Urea using 10 mM EPPS, pH 8.2, for overnight digestion with 1:50 (w/w) ratio of LysC (Wako Chemicals) at 37°C. Samples were further diluted to 1 M Urea and digested at 37°C for additional 6 h with 1:100 (w/w) ratio of sequencing grade Trypsin (Promega). Digests were acidified using trifluoroacetic acid to obtain pH < 3 and purified using 50 mg tC18 SepPak columns (Waters). Peptides were dried and resuspended in 0.2 M EPPS, pH 8.2, and 10% acetonitrile (ACN). Micro BCA protein assay kit (Thermo Fisher Scientific) was used to determine peptide concentration. 27 μg peptide per sample was labeled with 1:2.5 (w/w) ratio of TMTpro 16plex label reagent (Thermo Fisher Scientific). A bridge channel was prepared by pooling 3 μg from all 72 samples which were TMT-labeled together and split into six 27 μg samples for each plex. HeLa (ATCC CCL-2) digests cultured in non-SILAC or heavy SILAC DMEM for over 4 wk were used as noise and boost channels, respectively, as described previously (33). The boost channel was used at 2:1 M ratio (54 μg) compared with other samples. The labeling was organized such that all triplicates were spread across a different TMTpro 16plex resulting in three plexes for each virus and a total of six plexes comprising a noise channel (126), 12 samples (six controls and six infected; 127N-132C), a boost channel (133C) and a bridge channel (134N), each. The ratios between all channels were further normalized following a single injection measurement of each plex by LC-MS/MS which was also used to control and confirm the labeling efficiency (>99% labeling of all peptide sequences for all plexes). All samples were pooled in equimolar ratio within each plex and acidified before desalting and removal of excess TMT using tC18 SepPak columns (50 mg; Waters). Peptides were dried before fractionation.

### High pH reverse phase fractionation

The Dionex Ultimate 3000 analytical HPLC was used to perform high pH reverse phase fractionation. For each plex, 432 μg of pooled and purified TMT-labeled samples were resuspended in 10 mM ammonium-bicarbonate (ABC), 5% ACN, and separated on a 250 mm long C18 column (X-Bridge, 4.6 mm ID, 3.5-μm particle size; Waters) using a 70 min multistep gradient from 100% Solvent A (5% ACN, 10 mM ABC in water) to 60% Solvent B (90% ACN, 10 mM ABC in water). Eluting peptides were collected every 45 s. The resulting 96 fractions were cross-concatenated into 24 fractions and subsequently dried for liquid chromatography mass spectrometry (LC-MS) analysis.

### Mass spectrometry

5 μg of dried peptides of each fraction was resuspended in 2% (vol/vol) ACN/1% (vol/vol) formic acid (FA) solution and 1 μg was shot with settings described previously (33, 34). Data acquisition was performed using centroid mode on an Orbitrap Fusion Lumos mass spectrometer hyphenated to an easy-nLC 1200 nano HPLC system with a nanoFlex ion source (Thermo Fisher Scientific). A spray voltage 2.6 kV was applied with the transfer tube heated to 300°C and a funnel RF of 30%. Internal mass calibration was enabled (lock mass 445.12003 m/z). Peptides were separated on a self-made, 30 cm long, 75 μm ID fused-silica column, packed in-house with 1.9 μm C18 particles (ReproSil-Pur, Dr. Maisch) and heated to 50°C using an integrated column oven (Sonation). HPLC solvents consisted of 0.1% FA in water (Buffer A) and 0.1% FA, 80% ACN in water (Buffer B).

Individual peptide fractions were eluted by a nonlinear gradient from 7 to 40% B over 90 min followed by a step-wise increase to 90% B in 6 min and held for another 9 min. Full scan MS spectra (350–1,400 m/z) were acquired with a resolution of 120,000 at m/z 200, maximum injection time of 100 ms and AGC target value of 4 × 10^5^. The 10 most intense precursors with a charge state between 2 and 5 per full scan were selected together with their labeled counterparts (Targeted Mass Difference Filter, arginine and lysine δ mass, 5–100% partner intensity range with 7 ppm mass difference tolerance), resulting in 20 dependent scans (Top20). Precursors were selected with a quadrupole isolation window of 0.4 Th and fragmented by HCD with a normalized collision energy of 35%. MS2-analysis was performed in the Orbitrap with a resolution of 50,000 at m/z 200 using a maximum injection time of 86 ms and an AGC target value of 1 × 10^5^. To limit repeated sequencing of already acquired precursors a dynamic exclusion of 60 s and 7 ppm was set and advanced peak determination was deactivated.

### Data analysis

Raw files were analyzed using Proteome Discoverer (PD) 2.4 software (Thermo Fisher Scientific). Spectra were selected using default settings and database searches were performed using the Sequest HT node in PD against trypsin digested Homo Sapiens SwissProt database (20,531 sequences), SARS-CoV-2 database (UniProt pre-release, 14 sequences), MERS-CoV database (10 sequences), and MaxQuant contaminants FASTA. Static modifications were set as TMTpro at the N-terminus and carbamidomethyl at cysteine residues. Search was performed using Sequest HT taking the following dynamic modifications into account: TMTpro (K, +304.207 D), TMTpro+K8 (K, +312.221 D), and Arg10 (R, +10.008 D). Precursor mass tolerance was set to 10 ppm and fragment mass tolerance was set to 0.02 D. Default Percolator settings in PD were used to filter perfect spectrum matches (PSMs). Reporter ion quantifications were achieved using default settings in the consensus workflow. Minimal signal-to-noise ratio was set to 5. PSMs and protein files were exported for translatome and proteome analyses using in-house Python scripts (Python 3.7.1 and packages-pandas 0.23.4, numpy 1.15.4 and scipy 1.1.0) as described before (32). Briefly, for translatome, PSMs were adjusted with their ion injection time (IT) to account for peptide abundance in TMT intensities. Adjusted PSMs were normalized using total intensity normalization, followed by extraction of heavy labeled peptides and baseline correction using the noise channel where negative intensities were substituted with zero. All heavy peptides belonging to the same UniProt accession number were summed and combined with the protein file. For each set of the three plexes belonging to either SARS-CoV-2 or MERS-CoV, internal reference scaling (88) normalization was performed to obtain global translation rates across replicates. Proteome was quantified by IT adjustment of PSMs, concatenation of adjusted PSMs belonging to the same viral infection (three plexes) and processed exclusively for each viral infection using total intensity normalization, internal reference scaling, and trimmed mean of M-values (TMM (89)) normalization. Peptides belonging to the same UniProt accession were summed and global proteome quantifications for each virus were obtained. The mean log_2_ fold-changes were calculated for all quantified proteins in infected samples with respect to their corresponding controls (n = 3 independent biological replicates each), unless stated otherwise. Statistical significance was assessed using a two-sided, unpaired *t* test assuming equal variance, unless stated otherwise. All contaminants including all detected keratins were removed before further analysis.

### Data processing and software

#### PCA, hierarchical clustering, and viral profile plots

PCA, cluster analysis, and viral protein profile plot analyses were performed using Perseus software (version 1.6.10.50) (90). PCA was performed for each viral infection at the proteome and translatome level by using replicate average. For hierarchical clustering, all samples were filtered for proteins detected in both SARS-CoV-2 and MERS-CoV plexes. For each protein, the mean log_2_ fold-change upon infection compared with corresponding controls were used to perform row-wise clustering with Euclidean or Manhattan distance, average linkage and pre-processed with k-means (12 clusters and 10 iterations). Viral profile plots were analyzed for each cell type and viral infection, separately. First, all proteins were Z-score normalized and a reference profile was generated using all detected viral proteins at the translatome level. All protein profiles were compared with this reference profile using Pearson correlation for distance and false discovery rate computation.

#### Cluster analyses

Protein clusters were functionally annotated using DAVID Bioinformatics Resources 6.8 (91, 92) web-tool with a background of all quantified proteins of the study in each subset (translatome and proteome). Clusters of interest were selected and presented in more detail. Dot plots and ridge plots representing pathway enrichments and mitochondrial suborganellar localization, respectively, were created using RStudio (93) version 1.3.959 with packages–ggridges, ggplot2, dplyr, tidyr, forcats, stringr, and ggstance.

#### Network analyses and Venn diagram

Cytoscape (94) 3.8 software was used with StringApp (95) 1.5.1, BiNGO (96) 3.0.4 plugin for gene ontology analysis, OMICS visualizer (97) 1.3.0, and yFiles Layout Algorithms 1.1. For network and gene ontology analyses, gene sets were extracted from data as indicated using fold-change and significance cutoffs. Venn diagram was created using Bioinformatics and Evolutionary Genomics web-tool.

#### Pathway enrichment analyses

To identify proteins belonging to pathways of interest the following datasets were used–Immune system process (GO: 0002376), Renal pathology (GSEA datasets for AKI [HP:0001919], chronic kidney disease [HP:0012622], renal insufficiency [HP:0000083], GO-term renal system process [GO:0003014], renal tubular dysfunction [HP:0000124] and renal tubular atrophy [HP:0000092]), and the Human MitoCarta2.0 (98) was used for annotation of mitochondrial proteins and their suborganellar localization. Previously published (32), Caco-2 cell line proteomic dataset was used to compare immune response between immortalized cell line and primary cells. ISGs relevant for SARS-CoV-2 infection were extracted from Martin-Sancho et al (42), and compared with ISGs that were quantified in our dataset to compare overlap and differences across viruses. Cellular localization was determined using COMPARTMENTS subcellular localization database (99) accessed via GeneCards database (100). Only compartments with a score of ≥3 were included.

#### Statistical analyses

No statistical analyses were used to predetermine sample size. Protein significance was tested using unpaired two-sided *t* test with equal variance assumed. Global fold-change distribution’s mediation from 0 was tested using one-sample *t* test assuming normality based on Q–Q plots. Statistical analysis was performed using Microsoft Excel 2016 and OriginPro 2020b (101). For network and gene ontology analyses, all statistical computations were performed by the corresponding packages.

## Data Availability

The LC-MS/MS proteomics data have been deposited in the ProteomeXchange Consortium via the PRIDE (102) partner repository with the dataset identifier: PXD024398.

## Supplementary Material

Reviewer comments
